# Generation of Thyroid Tissues From Embryonic Stem Cells *via* Blastocyst Complementation *In Vivo*

**DOI:** 10.3389/fendo.2020.609697

**Published:** 2020-12-14

**Authors:** Qingsong Ran, Qiliang Zhou, Kanako Oda, Akihiro Yasue, Manabu Abe, Xulu Ye, Yingchun Li, Toshikuni Sasaoka, Kenji Sakimura, Yoichi Ajioka, Yasuo Saijo

**Affiliations:** ^1^ Department of Medical Oncology, Niigata University Graduate School of Medical and Dental Sciences, Niigata, Japan; ^2^ Department of Comparative and Experimental Medicine, Brain Research Institute, Niigata University, Niigata, Japan; ^3^ Department of Orthodontics and Dentofacial Orthopedics, Institute of Biomedical Sciences, Tokushima University Graduate School, Tokushima, Japan; ^4^ Department of Animal Model Development, Brain Research Institute, Niigata University, Niigata, Japan; ^5^ Division of Molecular and Diagnostic Pathology, Niigata University Graduate School of Medical and Dental Sciences, Niigata, Japan

**Keywords:** blastocyst complementation, embryonic stem cells, Fgf10, pluripotent stem cells, thyroid generation

## Abstract

The generation of mature, functional, thyroid follicular cells from pluripotent stem cells would potentially provide a therapeutic benefit for patients with hypothyroidism, but *in vitro* differentiation remains difficult. We earlier reported the *in vivo* generation of lung organs *via* blastocyst complementation in fibroblast growth factor 10 (*Fgf10*), compound, heterozygous mutant (*Fgf10* Ex1^mut^/Ex3^mut^) mice. Fgf10 also plays an essential role in thyroid development and branching morphogenesis, but any role thereof in thyroid organogenesis remains unclear. Here, we report that the thyroids of *Fgf10* Ex1^mut^/Ex3^mut^ mice exhibit severe hypoplasia, and we generate thyroid tissues from mouse embryonic stem cells (ESCs) in *Fgf10* Ex1^mut^/Ex3^mut^ mice *via* blastocyst complementation. The tissues were morphologically normal and physiologically functional. The thyroid follicular cells of *Fgf10* Ex1^mut^/Ex3^mut^ chimeric mice were derived largely from GFP-positive mouse ESCs although the recipient cells were mixed. Thyroid generation *in vivo via* blastocyst complementation will aid functional thyroid regeneration.

## Introduction

Continuous, oral thyroid hormone replacement therapy is indispensable for patients with hypothyroidism caused by total thyroidectomy or etiological factors. Although this is relatively simple, effective, safe, and inexpensive, it can be difficult to maintain the complex homeostatic interactions of various hormones (1, 2), and the side-effects of over-replacement include cardiac events and osteoporosis also cannot be ignored (2, 3). Regeneration and transplantation of thyroid tissue to physiologically supplement thyroid hormone levels is an alternative (radical) treatment strategy (4, 5). Derivation of thyroid follicular cells *via* directed differentiation of pluripotent stem cells (PSCs) *in vitro*, using growth factor-supplemented media, failed to regenerate mature thyroid follicular cells expressing the full genetic suite required for functional thyroid hormone biosynthesis (6–10). Using an embryonic stem cell (ESC) line hosting a GFP reporter-linked cDNA targeting the locus encoding the homeodomain-containing thyroid transcription factor 1 (TTF1 or Nkx2-1), Kurmann et al. reported the generation of functional thyrocytes *via* activation of bone morphogenetic protein (Bmp) and fibroblast growth factor (Fgf) signaling *in vitro (* 11). Alternatively, transient forced overexpression of the transcription factors TTF1 and Paired box gene 8 (Pax8) of mouse or human ESCs allowed the cells to differentiate into functional thyroid follicular cells *in vitro *(12–15). However, the problems associated with *in vitro* generation of mature thyroid follicular tissue from PSCs, including low differentiation efficiency, the need for genetic labeling to sort and enrich progenitors, and the risk of tumor formation from undifferentiated PSCs after transplantation, limit the clinical applications of cell therapy.

Recently, *in vivo* models of organ generation *via* blastocyst complementation have shown promise. Generation of the pancreas (16, 17), kidney (18, 19), blood vasculature (20) and lung (21) *via* intra- or inter-species blastocyst complementation have been reported. Very recently, we used fibroblast growth factor 10, (*Fgf10*), compound, heterozygous mutant (*Fgf10* Ex1^mut^/Ex3^mut^) mice to generate lungs *via* blastocyst complementation (22). *Fgf10* Ex1^mut^/Ex3^mut^ mice exhibited limb and lung deficiencies, as did *Fgf10* Ex1−/− and *Fgf10* Ex3−/− mice, as well as other *Fgf10*-knockout mice (23–25). Complementation with ESCs enabled *Fgf10* Ex1^mut^/Ex3^mut^ mice to survive to adulthood without any abnormality.

In contrast to the relatively distinct role played by Fgf10 in lung development and branching morphogenesis (23, 24, 26–28), indefiniteness remains in thyroid organogenesis. Thyroid agenesis has been reported in mice deficient in Fgf10 (24) or its receptor Fgfr2b (29), indicating that Fgf10–Fgfr2b signaling plays a crucial role in thyroid organogenesis. However, although the thyroid primordium was absent at E13, the stage at which thyroid morphogenesis was impaired was not explored. Nkx2-1^+^/Sox 9^+^ thyroid progenitors were detected in the thyroid placode at E9.5; weak expression of Fgfr2b in the thyroid primordium at E12.5; and distinct expression of Fgf10 in the mesenchyme at E15.5 (30). By contrast, it has been reported that *Fgf10*-null mutant mouse embryos did not exhibit thyroid agenesis but rather severe hypoplasia (the thyroid was shaped normally) (30, 31). Similarly, conditional knockout of *Fgf10* (*Wnt1cre Fgf10* fl/fl) in neural crest, from which several head tissues are derived (including the mesenchyme around the developing thyroid glands), resulted thyroid remnants (31). Therefore, we explored the thyroid phenotype of *Fgf10* Ex1^mut^/Ex3^mut^ mice and the possibility of thyroid generation in such mice from PSCs (thus *via* blastocyst complementation).

Here, we report that the thyroids of *Fgf10* Ex1^mut^/Ex3^mut^ mice are normally shaped but severely hypoplastic. Complementation with ESCs rescued thyroid organogenesis. Generation of thyroids *in vivo via* blastocyst complementation will aid functional thyroid regeneration.

## Materials and Methods

### Generation of Fgf10 Ex1^mut^/Ex3^mut^ Mice and Chimeric Mice

All animal experiments were approved by the Institutional Animal Care and Use Committee of Niigata University, Niigata, Japan (approval number SA00233). *Fgf10* Ex1^wild/mut^ and *Fgf10* Ex3^wild/mut^ mice were generated using the CRISPR/Cas9 system as described in our previous report (22). *Fgf10* Ex1^mut^/Ex3^mut^ mice were obtained by intercrossing *Fgf10* Ex1^wild/mut^ mice with *Fgf10* Ex3^wild/mut^ mice. Generation of *Fgf10* Ex1^mut^/Ex3^mut^ chimeric mice *via* blastocyst complementation proceeded as described previously (22). Briefly, embryos were prepared *via in vitro* fertilization of *Fgf10* Ex3−/+ ova with *Fgf10* Ex1−/+ sperm, and five to eight GFP-expressing mouse RENKA C57BL/6NCrlCrlj ESCs (#CFS-EGFP27; Brain Research Institute, Niigata University) were prepared and microinjected into the perivitelline space of eight-cell/morula-stage embryos. After further culture *in vitro*, the embryos were transferred into the uteri of pseudopregnant, recipient ICR female mice. Genotyping of the *Fgf10* Ex1^mut^/Ex3^mut^ mice and chimeric mice were performed using the Surveyor System and DNA sequencing, as described previously (22).

### Histological Analysis

Mouse tissues were fixed in 10% (v/v) neutral buffered formalin, embedded in paraffin, sectioned, and the sections deparaffinized with xylene and hydrated in a graded series of ethanol baths. Hematoxylin and eosin (H&E) and immunoﬂuorescence staining were performed as described previously (22). The primary antibodies were anti-GFP polyclonal antibody (goat IgG, 1:200; #GTX26673; GeneTex, Irvine, CA, USA); anti-TTF1 monoclonal antibody (rabbit IgG, 1:200; #ab76013; Abcam, Cambridge, UK); anti-FOXE1 polyclonal antibody (rabbit IgG, 1:200; #bs-0446r; Bioss, Woburn, MA, USA); anti-Pax8 antibody (rabbit IgG, 1:200; #10337-1-AP; Proteintech, Chicago, IL, USA); anti-thyroglobulin monoclonal antibody (rabbit IgG,1:200; #ab156008; Abcam); anti-T3 polyclonal antibody (rabbit IgG, 1:200; #MBS2001953; MyBioSource, San Diego, CA, USA); anti-calcitonin polyclonal antibody (rabbit IgG, 1:200; #GTX134005; GeneTex); anti-vimentin monoclonal antibody (rabbit IgG, 1:200; #ab92574; Abcam); and anti-Ki-67 polyclonal antibody (rabbit IgG, 1:200; #ab15580, Abcam). Donkey anti-goat IgG-Alexa Fluor 488 (1:200; #A11055; Invitrogen, Carlsbad, CA, USA) and donkey anti-rabbit IgG-Alexa Fluor 594 (1:200; #A21207; Invitrogen) served as secondary antibodies. Nuclei were counterstained with 4′,6-diamidino-2-phenylindole (DAPI) and fluorescence images acquired using a C1si confocal microscope (Nikon, Tokyo, Japan).

TTF1-positive cells were counted in over 1,500 cells in at least three images (200× magnification) randomly selected from the thyroids of each mouse. GFP-positive cells among TTF1-positive cells were counted and the percentage of GFP/TTF1-positive cells was then calculated.

### Contrast-Enhanced Micro-Computed Tomography

To explore the macroscopic phenotypes of the thyroid tissues of *Fgf10* Ex1^mut^/Ex3^mut^ neonatal mice and *Fgf10* Ex1^mut^/Ex3^mut^ chimeric neonatal mice, contrast-enhanced micro-CT analysis was performed as described previously (22) with slight modifications. Briefly, neonatal mice were first fixed in 4% (v/v) paraformaldehyde at 4°C for 2 days. A midline cervical incision was then created and the larynx, trachea, and thyroid exposed. Then, the mice were immersed in 25% (v/v) Lugol’s iodine solution at room temperature for 5 days. Subsequently, the samples were scanned using a micro-CT device (Nittetsu Elex, Tokyo, Japan) and the data analyzed with the aid of TRI/3D-Bon software (Ratoc System Engineering Co. Ltd., Tokyo, Japan).

### Enzyme-Linked Immunosorbent Assays

Serum tri-iodothyronine (T3) and thyroxine (T4) concentrations were measured using ELISA kits (CSB-E05086m for T3, CSB-E05083m for T4; CUSABIO, Wuhan, China), according to the manufacturer’s protocols. Briefly, 50 µl of standards or blood samples was added to 96-well plates, followed by 50 µl of conjugate reagents; incubation proceeded for 60 min at 37°C. The liquid was aspirated, the wells washed three times, 50 µl of the HRP–avidin reagent added, and the plates incubated for 30 min at 37°C. The liquid was aspirated, the wells washed three times, and 50 µl of substrates A and B added. After incubation for 15 min at 37°C in the dark, 50 µl of stop solution was added and the optical density at 450 nm measured within 10 min using a microplate reader. All tests were performed in duplicate.

### Statistical Analysis

Data are presented as the means ± standard deviations. One-way analysis of variance and the Tukey–Kramer test were used to assess the significance of differences. A *p*-value <0.05 was deemed to indicate significance.

## Results

### *Fgf10* Ex1^mut^/Ex3^mut^ Mice Exhibit Severe Thyroid Hypoplasia

**Figure 1:**
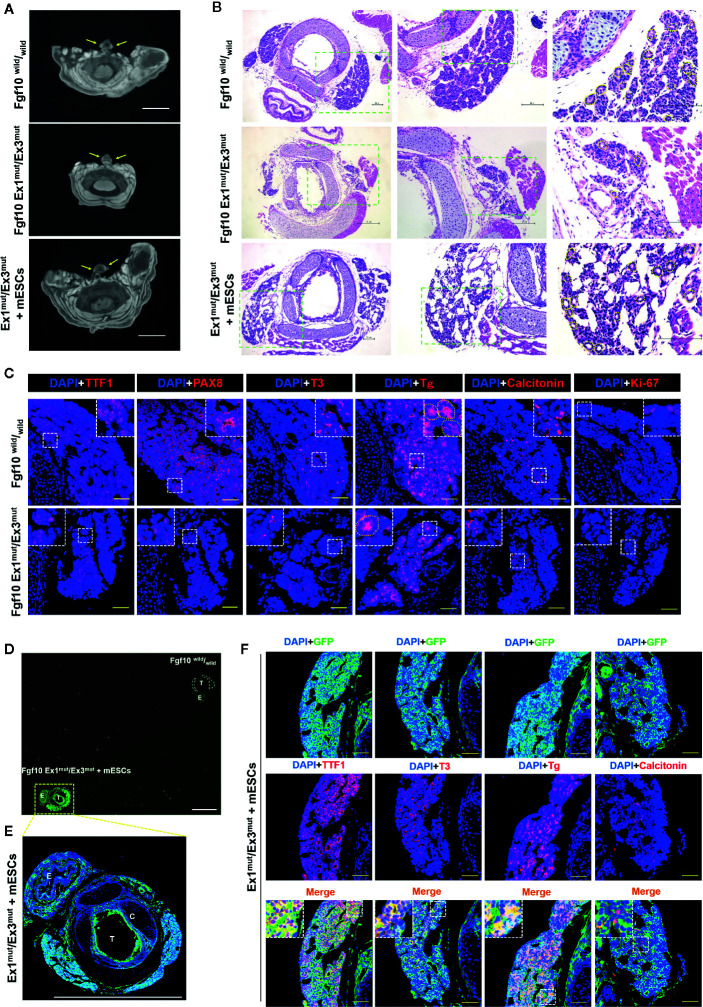
Characterization of the thyroids of *Fgf10* Ex1^mut^/Ex3^mut^ neonates and Ex1^mut^/Ex3^mut^ chimeric neonates complemented with mouse embryonic stem cells (mESCs). **(A)** Axial micro-computed tomography images of the neck regions of *Fgf10* ^wild^/_wild_ and Ex1^mut^/Ex3^mut^ neonates and Ex1^mut^/Ex3^mut^ chimeric neonates. Yellow arrows indicate thyroid lobes adjacent to the tracheae. Scale bar = 2 mm. **(B)** Hematoxylin and eosin staining of cervical cross-sections of *Fgf10* ^wild^/_wild_ and Ex1^mut^/Ex3^mut^ neonates and Ex1^mut^/Ex3^mut^ chimeric neonates. The right panels show magnified views of the areas indicated by the green dotted lines in the left panels. Scale bars = 100 μm. **(C)** Immunofluorescence staining of the thyroids of *Fgf10* ^wild^/_wild_ and Ex1^mut^/Ex3^mut^ neonates for various markers (red): TTF1, thyroid transcription factor1; PAX8, paired box gene 8; T3, tri-iodothyronine; Tg, thyroglobulin; Calcitonin and Ki-67. Nuclei were stained with DAPI (blue). Scale bars = 50 μm. Yellow dotted lines in **(B**, **C)** indicated representative thyroid follicles. **(D–F)** Immunofluorescence staining of the thyroid of an *Fgf10* Ex1^mut^/Ex3^mut^ chimeric neonate. **(D)** A low magnification image acquired using a stereo fluorescence microscope. The thyroid of an *Fgf10* ^wild^/_wild_ neonate placed on the same slide served as the control. White dotted lines indicate the thyroid glands. **(E)** Image acquired using a confocal microscope (with slight magnification) of the tissue indicated by the yellow dotted box in **(D)**. Scale bars = 1 mm. T, trachea; C, cartilage; E, esophagus. **(F)** Immunofluorescence staining of the thyroids of *Fgf10* Ex1^mut^/Ex3^mut^ neonates for GFP (green) and thyroid markers (red): TTF1, T3, Tg and Calcitonin. Nuclei were stained with DAPI (blue). Insets in **(C**, **E)** show magnified views of the areas indicated with white dotted lines. Scale bars = 50 μm.

*Fgf10* Ex1^mut^/Ex3^mut^ mice were generated as previously reported (22). Consistent with the data of a recent study on embryonic growth of the thyroid gland in *Fgf10*-null mutant mice (30), neonatal *Fgf10* Ex1^mut^/Ex3^mut^ mice exhibited bilateral thyroid remnants (**Figure 1A**) on micro-CT analysis. Serial sections of the entire glands (n = 5) confirmed that the thyroids were normally shaped but smaller than those of *Fgf10* ^wild^/_wild_ neonates (**Supplemental Videos 1 and 2**). H&E and immunofluorescence staining indicated that the hypoplastic thyroids glands of *Fgf10* Ex1^mut^/Ex3^mut^ mice had a lower proportion of parenchyma, decreased branching, and fewer follicles than normal mouse thyroids (**Figures 1B, C**). Immunofluorescence staining indicated that the number of thyroid cells expressing TTF1 and Pax8 (the most important transcription factors in terms of thyroid gland organogenesis) was decreased in neonatal *Fgf10* Ex1^mut^/Ex3^mut^ mice compared to neonatal *Fgf10* ^wild^/_wild_ mice (**Figure 1C**). Although the protein levels seem to be similar, the total expression levels of thyroglobulin (Tg) (a precursor protein of thyroid hormone) and tri-iodothyronine (T3) were reduced in neonatal *Fgf10* Ex1^mut^/Ex3^mut^ mice (**Figure 1C**). Ki-67 positive proliferating cells were obviously reduced in thyroids of neonatal *Fgf10* Ex1^mut^/Ex3^mut^ mice compared to neonatal *Fgf10* ^wild^/_wild_ mice (**Figure 1C**). The expression of calcitonin in the neonatal *Fgf10* Ex1^mut^/Ex3^mut^ mice did not seem to decrease significantly (**Figure 1C**), in agreement with a previous report that Fgf10 is not involved in parafollicular cell differentiation (30).

### Generation of Thyroid Tissues in *Fgf10* Ex1^mut^/Ex3^mut^ Mice

We next sought to generate thyroid tissues from PSCs in *Fgf10* Ex1^mut^/Ex3^mut^ mice *via* blastocyst complementation. Micro-CT confirmed the existence of thyroids adjacent to the trachea at the front of the neck of neonatal *Fgf10* Ex1^mut^/Ex3^mut^ chimeras; the glands were of normal shape and size (**Figure 1A**). The thyroids of *Fgf10* Ex1^mut^/Ex3^mut^ chimeric neonates (**Figure 1B** and **Supplemental Video 3**) were histologically normal (thus similar to those of *Fgf10* ^wild^/_wild_ neonates) (**Figure 1B** and **Supplemental Video 1**). The thyroid tissues of *Fgf10* Ex1^mut^/Ex3^mut^ chimeras exhibited high-level GFP expression compared to those of *Fgf10* ^wild^/_wild_ neonates (**Figures 1D, E**), indicating a major contribution from GFP-expressing mouse ESCs. The levels of TTF1, Tg, and T3 in the thyroids of neonatal *Fgf10* Ex1^mut^/Ex3^mut^ chimeras (**Figure 1F**) were similar to those of neonatal *Fgf10* ^wild^/_wild_ mice (**Figure 1C**). The GFP expression of TTF1-positive follicular cells predominated but was mosaic, while those of calcitonin-positive parafollicular cells and vimentin-positive stromal cells showed no preponderance (**Figure 1F**). These data indicated that thyroid tissues were generated in *Fgf10* Ex1^mut^/Ex3^mut^ mice *via* blastocyst complementation.

### Characterization of the Thyroids of Adult *Fgf10* Ex1^mut^/Ex3^mut^ Chimeric Mice

**Figure 2:**
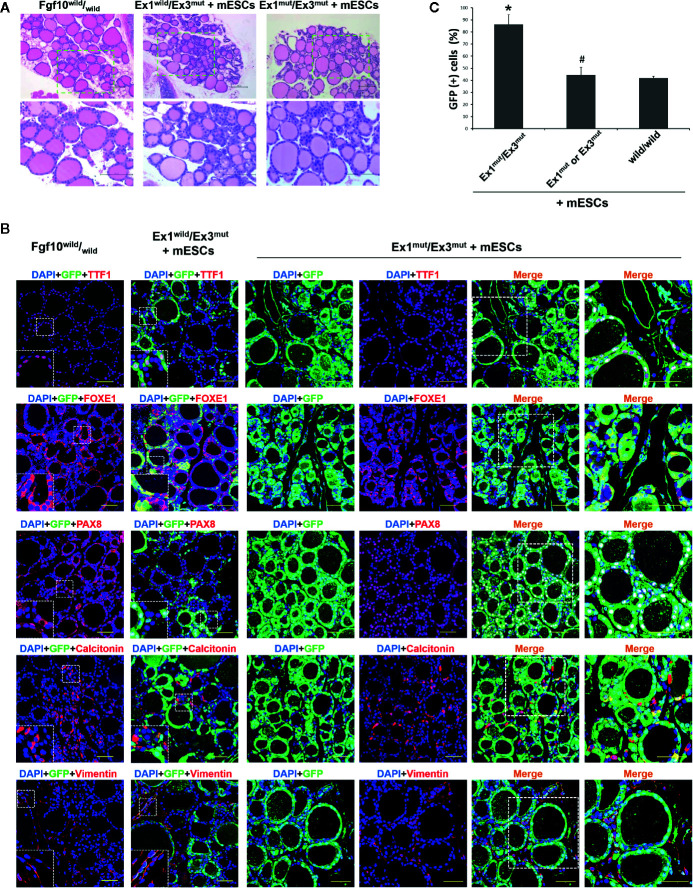
Characterization of the thyroids of adult *Fgf10* Ex1^mut^/Ex3^mut^ chimeric mice complemented with mouse embryonic stem cells (mESCs). **(A)** Hematoxylin and eosin staining of thyroid tissues from adult *Fgf10* ^wild^/_wild_ mice, and *Fgf10* Ex1^wild^/Ex3^mut^ and Ex1^mut^/Ex3^mut^ chimeric mice. The bottom panels show magnified views of the areas indicated by the dotted lines in the top panels. **(B)** Immunofluorescence staining of the thyroids of Fgf10 Ex1^mut^/Ex3^mut^ chimeric neonates for GFP (green) and various markers (red): TTF1, thyroid transcription factor 1; FOXE1, forkhead box E1; and PAX8, paired box gene 8 for follicular cells, Calcitonin for parafollicular cells, and Vimentin for stromal cells. Nuclei were stained with DAPI (blue). *Fgf10* ^wild^/_wild_ and *Fgf10* Ex1^wild^/Ex3^mut^ chimeric mice served as controls. Insets show magnified views of the areas indicated with white dotted lines. The right panels show magnified views of the areas indicated by the dotted lines in the left panels. Scale bars = 100 μm for **(A)**; 50 μm for **(B)**. **(C)** Enumeration of GFP/TTF1-positive thyroid follicular cells in adult *Fgf10* Ex1^mut^/Ex3^mut^, *Fgf10* Ex1^mut^ or Ex3^mut^, and *Fgf10* ^wild^/_wild_ chimeric mice. Data are expressed as the means ± standard deviations; n = 3/group. *p < 0.05 *versus* other treatments; ^#^ *p* > 0.05 *versus Fgf10* ^wild^/_wild_ chimeras.

We showed that survival of *Fgf10* Ex1^mut^/Ex3^mut^ mice to adulthood was rescued by complementation with mouse ESCs (22). Next, we analyzed the thyroid tissues of five *Fgf10* Ex1^mut^/Ex3^mut^ adult chimeric mice. We lacked data on adult *Fgf10* Ex1^mut^/Ex3^mut^ mice because they died immediately after birth; they had no lungs. The low proportion of parenchyma in the thyroids of *Fgf10* Ex1^mut^/Ex3^mut^ neonates (**Figure 1B**) recovered in the thyroid tissues of adult *Fgf10* Ex1^mut^/Ex3^mut^ chimeras (**Figure 2A**). The thyroid follicles of adult *Fgf10* Ex1^mut^/Ex3^mut^ chimeras were well-organized spheres lined with follicular cells surrounding the lumina that contained a colloid, as in adult *Fgf10* ^wild^/_wild_ mice (**Figure 2A**). The thyroid follicular cells of adult *Fgf10* Ex1^mut^/Ex3^mut^ chimeras expressed TTF1, FOXE1 (formerly TTF2), and Pax8 at levels similar to those of adult *Fgf10* ^wild^/_wild_ mice (**Figure 2B**). Calcitonin-positive parafollicular cells were detected in connective tissue adjacent to the thyroid follicles, as in adult *Fgf10* ^wild^/_wild_ mice (**Figure 2B**). Thus, the thyroids of adult *Fgf10* Ex1^mut^/Ex3^mut^ chimeric mice were histologically normal.

**Figure 3:**
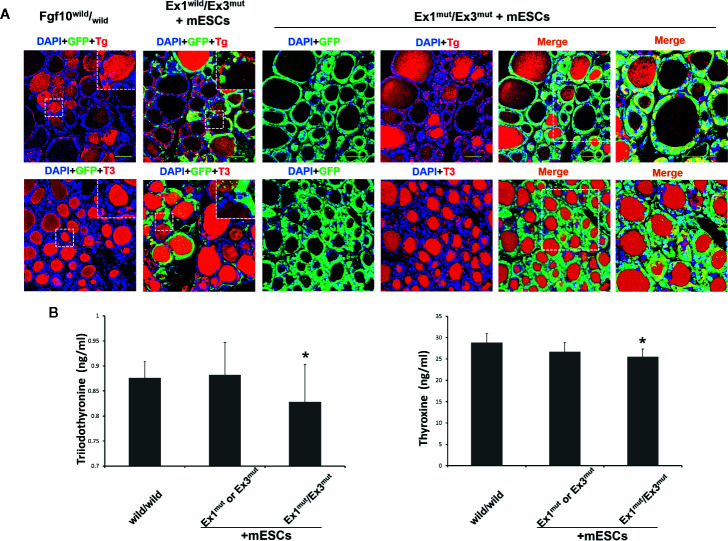
*In vivo* thyroid functionality assessment in adult *Fgf10* Ex1^mut^/Ex3^mut^ chimeric mice complemented with mouse embryonic stem cells (mESCs). **(A)** Immunofluorescence staining of the thyroids. Thyroid follicles were analyzed by staining for GFP (green) and markers of thyroid function. Tg, thyroglobulin; T3, tri-iodothyronine. Insets show magnified views of the areas indicated with white dotted lines. The right panels show magnified views of the areas indicated by dotted lines in the left panels. Nuclei were stained with DAPI (blue). *Fgf10* ^wild^/_wild_ and *Fgf10* Ex1^wild^/Ex3^mut^ chimeric mice served as controls. Scale bar = 50 μm. **(B)** ELISA analyses of serum tri-iodothyronine (T3) and thyroxine (T4) concentrations in adult *Fgf10* Ex1^mut^/Ex3^mut^ chimeric mice. Adult *Fgf10* ^wild^/_wild_ and *Fgf10* Ex1^mut^ or Ex3^mut^ chimeric mice served as controls. Data are expressed as the means ± standard deviations; n = 3 for the adult *Fgf10* Ex1^mut^/Ex3^mut^ chimeric mouse group and n = 4 for the adult *Fgf10* ^wild^/_wild_ and *Fgf10* Ex1^mut^ or Ex3^mut^ chimeric mouse groups; *p > 0.05 *versus* other treatments.

Next, we investigated the contribution of GFP-expressing mouse ESCs to the thyroids. Extremely strong, diffuse, GFP expression across all thyroid tissues was observed in *Fgf10* Ex1^mut^/Ex3^mut^ adult chimeras compared to adult *Fgf10* ^wild^/_wild_ mice or *Fgf10* Ex1^wild^/Ex3^mut^ chimeras (**Figure 2B**). In *Fgf10* Ex1^mut^/Ex3^mut^ adult chimeric mice, large proportions of the TTF1-, FOXE1-, and Pax8-positive follicular cells were GFP-positive, indicating that the cells were derived principally from mouse ESCs (**Figure 2B**). The extent of GFP expression in non-follicular regions, including parafollicular cells, blood vessels, and connective tissues, did not differ between the *Fgf10* Ex1^mut^/Ex3^mut^ and Ex1^wild^/Ex3^mut^ chimeras (**Figure 2B**). Moreover, 86.4 ± 7.9% of follicular cells in adult *Fgf10 Ex1^mut^/Ex3^mut^* chimeras were derived from GFP-positive mouse ESCs, a greater proportion than in adult *Fgf10* ^wild^/_wild_ and Ex1^mut^ or Ex3^mut^ chimeras (**Figure 2C**). Next, we assessed the physiological function of the thyroid tissues of adult *Fgf10* Ex1^mut^/Ex3^mut^ chimeras. Immunofluorescence staining confirmed cytosolic expression of Tg and deposition thereof in the thyroid follicular lumina (**Figure 3A**). T3 was also detected in the colloid, as in adult *Fgf10* ^wild^/_wild_ mice (**Figure 3A**). ELISA confirmed that the plasma T3 and thyroxine (T4) levels of adult *Fgf10* Ex1^mut^/Ex3^mut^ chimeras were similar to those of adult *Fgf10* ^wild^/_wild_ mice and Ex1^mut^ or Ex3^mut^ chimeric mice (**Figure 3B**). Thus, thyroids of adult *Fgf10* Ex1^mut^/Ex3^mut^ chimeras were functional. Thus, the functional thyroid follicles were generated principally from mouse ESCs in adult *Fgf10* Ex1^mut^/Ex3^mut^ chimeric mice, *via* blastocyst complementation.

## Discussion

We generated thyroid tissues in *Fgf10* Ex1^mut^/Ex3^mut^ mice with severely hypoplastic thyroids *via* blastocyst complementation with mouse ESCs. The generated thyroids were morphologically normal and physiologically functional compared to those of *Fgf10* ^wild^/_wild_ mice. The generated thyroid tissues exhibited significant contributions from GFP-positive ESCs but the recipient cells were mixed.

Early during mouse thyroid development, thyroid progenitors expressing a specific combination of four critical transcription factors [Nkx2-1, Pax8, FOXE1 (Forkhead Box E1), and HHEX (hematopoietically expressed homeobox)] assemble to form the thyroid bud in the anterior foregut endoderm (32, 33). These transcription factors are linked to an integrated regulatory network that controls thyroid survival and migration during organogenesis, *via* cell-autonomous mechanisms (32, 33). Deletion of a gene encoding any of these transcription factors triggers athyreosis or severe thyroid hypoplasia (34). Fgf10 plays essential roles in the development of many organs such as the thyroid, limbs, lungs, and pituitary and salivary glands, mediated principally *via* the mesenchymal–epithelial interaction signaled through the receptor Fgfr2-IIIb (24, 35). Mice deficient in Fgf10 or Fgfr2b exhibit athyreosis, indicating that Fgf10 is required for thyroid budding and branching morphogenesis (24, 29). However, a recent study reported that most *Fgf10*-null mouse embryos exhibited small, unilateral remnant thyroids, indicating that organogenesis proceeded even in the complete absence of Fgf10 (31). Conditional, neural crest *Fgf10* knock-out reduced thyroid size to a lesser degree than in the null mutant, suggesting that a source of Fgf10 apart from the neural crest might be available to assist thyroid development (31). A recent work on thyroid branching morphogenesis showed that normally shaped, symmetrical thyroids were present in *Fgf10*-null mutant mouse embryos, but were severely hypoplastic (30). Fgf10–Fgfr2b signaling may thus be dispensable in terms of thyroid specification and early development, but is required to regulate organogenesis (30). We found that the thyroids of neonatal, *Fgf10* compound heterozygous mutant (*Fgf10* Ex1^mut^/Ex3^mut^) mice were severely hypoplastic but symmetrically residual, supporting the above observations in mouse embryos (30, 31). Furthermore, complementation with *Fgf10*wild-type ESCs rescued thyroid organogenesis both histologically and functionally in *Fgf10* Ex1^mut^/Ex3^mut^ mice, indicating that Fgf10 played essential roles in late thyroid development and organogenesis.

Although Fgf10 seems to be dispensable in terms of thyroid specification and early thyroid development, Fgf10-induced branching growth has been reported to account for over 80% of thyroid enlargement before birth (30). Given the symmetrical, severe thyroid hypoplasia of *Fgf10* Ex1^mut^/Ex3^mut^ mice, we expected that it might be possible to generate functional thyroid tissues from PSCs in such mice *via* blastocyst complementation. Indeed, high proportions of the thyroid follicular cells of *Fgf10* Ex1^mut^/Ex3^mut^ adult chimeric mice were GFP-positive (**Figure 2B**), indicating major contributions from donor ESCs. Localized Fgf10 expression by donor ESCs in the mesenchyme around developing thyroid glands would act non-selectively (*via* Fgfr2-IIIb-mediated mesenchymal–epithelial interaction signaling) on both GFP-positive donor cells (*Fgf10* ^wild^/_wild_) and GFP-negative host cells (*Fgf10* Ex1^mut^/Ex3^mut^) resident in the endoderm. However, other mechanisms [such as ectopic expression of Fgf10 in the GFP-positive donor epithelium (*Fgf10* ^wild^/_wild_), as indicated during lung generation *via* blastocyst complementation] (22) may explain in the relative preponderance of GFP-positive donor ESCs during thyroid development compared to the level in the *Fgf10* Ex1^mut^/Ex3^mut^ host epithelium. Importantly, ESC-derived thyroid follicles expressed and deposited T3 as did adult *Fgf10* ^wild^/_wild_ mice (**Figure 3A**). These data, together with the ELISA results indicating that adult *Fgf10* Ex1^mut^/Ex3^mut^ chimeras had normal T3 and T4 plasma levels compared to adult *Fgf10* ^wild^/_wild_ mice (**Figure 3B**), indicated that the mature, functional thyroid follicle tissues of adult *Fgf10* Ex1^mut^/Ex3^mut^ chimeras were generated predominantly from ESCs.

Directed *in vitro* differentiation of PSCs using growth factors has been reported, but failed to regenerate mature thyroid follicular cells (6–10). Derivation of functional thyroid follicular cells *in vitro* from mouse and human induced PSCs (4, 11), mouse ESCs (12, 13, 15), and human ESCs (14) has been reported using several protocols. However, the generation of such cells from PSCs is inefficient; enrichment and sorting of precursor cells currently requires genetic editing (TTF1 and Pax8 overexpression or labeling of targeted alleles) (34, 36). Also, the risk of tumor formation from undifferentiated PSCs on transplantation after *in vitro* differentiation cannot be ignored. Our current work indicates that mature, functional thyroid follicular cells can be generated from PSCs *via* blastocyst complementation. Although the generated thyroid tissues in *Fgf10* Ex1^mut^/Ex3^mut^ chimeras were mixtures of donor and host cells, this is not an argument against thyroid regeneration, because transplantation of mature thyroid follicular cells (not the organ) would suffice as therapy for patients with hypothyroidism. Sorting of PSC-derived mature follicular cells or follicular tissues is required. Furthermore, the low efficiency of adult *Fgf10* Ex1^mut^/Ex3^mut^ chimera generation (5 adult compound heterozygous chimeras weaned from 76 neonatal chimeras obtained by transplantation of 638 blastocysts) (22) and the undesirable thyroid chimerism of the present study require attention. The use of a conditional knockout method or other knockout targets such as Nkx2-1, Pax8, or Fgf2 (all of which are essential for early thyroid development) might be useful. Wen *et al*. recently generated lung and thyroid epithelial cell lineages almost entirely from mouse ESCs in Nkx2-1 knockout mice *via* blastocyst complementation (37). Exploring the possibility of generation of PSC-derived thyroid tissues *via* inter-species blastocyst complementation in rodents or livestock remains to be investigated (22). Another concern is that human PSCs-derived cells will appear in the brains and gonads of livestock, especially when generating human organs from PSCs in livestock using the current inter-species blastocyst complementation technique. The use of committed stem or progenitor cells, or PSCs genetically modified to restrict their differentiation potential, would address this issue (16), but clinical application remains some way off.

In summary, we showed that Fgf10 played an essential role in thyroid development and that thyroid tissues generated in thyroid hypoplastic *Fgf10* Ex1^mut^/Ex3^mut^ mice were largely derived from mouse ESCs *via* blastocyst complementation. Generation of PSC-derived thyroid tissues *via* blastocyst complementation is a promising approach to thyroid regeneration.

## Data Availability Statement

The original contributions presented in the study are included in the article/**Supplementary Material**. Further inquiries can be directed to the corresponding author.

## Ethics Statement

The animal study was reviewed and approved by Institutional Animal Care and Use Committee of Niigata University.

## Author Contributions

QR performed experiments, contributed to data analysis and interpretation, and assisted with manuscript preparation. KO and TS performed the embryo manipulation and animal experiments, and contributed to the analysis and interpretation of mouse data. AY generated the *Fgf10* knockout mouse and contributed to DNA analysis. MA and KS prepared the GFP-positive mouse ESCs and assisted with embryo manipulation. XY and YL performed some of the experiments. YA contributed to histological analysis and sequencing. YS and QZ designed the project, performed some of the experiments, analyzed the data, and wrote the manuscript. All authors contributed to the article and approved the submitted version.

## Funding

This work was supported in part by the Japan Society for the Promotion of Science (KAKENHI; grant nos. 18K15921 and 18H02817G).

## Conflict of Interest

The authors declare that the research was conducted in the absence of any commercial or financial relationships that could be construed as a potential conflict of interest.
