# Supplementary material for: Generation of Thyroid Tissues From Embryonic Stem Cells via Blastocyst Complementation In Vivo
Source: Front Endocrinol (Lausanne). 2020 Dec 14;11:609697. doi: 10.3389/fendo.2020.609697 (PMC7767966; doi:10.3389/fendo.2020.609697)
Supplement: Supplementary file 1 [file DataSheet_1.pdf]

Supplemental video 1. Continuous hematoxylin and eosin staining of serial sections of the thyroid gland of an *Fgf10* Ex1^{wild}/Ex3^{wild} neonate.

Supplemental video 2. Continuous hematoxylin and eosin staining of serial sections of the thyroid gland of an *Fgf10* Ex1^{mut}/Ex3^{mut} neonate with severe thyroid hypoplasia.

Supplemental video 3. Continuous hematoxylin and eosin staining of serial sections of the thyroid gland of an *Fgf10* Ex1^{mut}/Ex3^{mut} chimeric neonate complemented with embryonic stem cells.
